# Effectiveness of Constraint induced movement therapy as compared to bimanual therapy in Upper motor function outcome in child with hemiplegic Cerebral palsy

**DOI:** 10.12669/pjms.321.8491

**Published:** 2016

**Authors:** Hira Zafer, Imran Amjad, Arshad Nawaz Malik, Enfall Shaukat

**Affiliations:** 1 Dr. Hira Zafer, DPT, Riphah College of rehabilitation sciences, Riphah International University, Islamabad, Pakistan; 2 Dr. Imran Amjad, DPT, Riphah College of rehabilitation sciences, Riphah International University, Islamabad, Pakistan; 3 Dr. Arshad Nawaz Malik, DPT, Riphah College of rehabilitation sciences, Riphah International University, Islamabad, Pakistan; 4 Dr. Enfall Shaukat, DPT, Riphah College of rehabilitation sciences, Riphah International University, Islamabad, Pakistan

**Keywords:** Constraint induced movement therapy, Bimanual therapy, Hemiplegic Cerebral Palsy

## Abstract

**Objective:**

This study aims at determining the effectiveness of constraint induced movement therapy as compared to bimanual therapy for improving functional status in children with hemiplegic cerebral palsy.

**Methods:**

This study was a randomized control trial, children (n = 20) with spastic hemiplegic cerebral palsy was randomly allocated to CIMT (constraint induced movement therapy) and BMT (bimanual therapy) group. The children with spastic hemiplegia, age between 1.5 and 12 year and having 10 degrees of wrist extension and 10 degrees of finger extension were included in study. Treatment regime was two hours of daily training six days a week for two weeks. Constraint was applied to CIMT group for six hours. The outcome tool QUEST was used for baseline and post treatment assessment.

**Result:**

CIMT had superior outcome as compared to BMT in improving functional status (p=0.007). On QUEST tool grasp and dissociated movements results were significant (p=0.005) and (p=0.028) respectively. Weight bearing and protective extension resulted in no significant outcome (p=0.080) and (p=0.149) respectively. Dissociated movements and grasp are significantly improved but there is no difference for weight bearing and protective extension in CIMT treated group as compared to BMT treated group.

**Conclusion:**

CIMT approach is better in improving functional status of child with cerebral palsy as compared to BMT. Significant improvement in grasp and dissociated movement is noted in group of CIMT while there was no significant improvement in weight bearing and protective extension in CIMT group when compared to BMT. CIMT is considered the appropriate treatment approach for unilateral conditions while BMT for bilateral conditions.

## INTRODUCTION

Cerebral palsy is defined as “a group of permanent disorders of the development of movement and posture, causing activity limitations, which are attributed to non-progressive disturbances that occurred in the developing fetal or infant brain”. The motor disorders of cerebral palsy are often accompanied by disturbances of sensation, perception, cognition, communication, and behavior and by secondary musculoskeletal problem”.^1^ There are different risk factor associated with the cerebral palsy after birth including of low APGAR score, less weighed placenta, respiratory problems, infection and seizures in neonates.^2^ In literature there are multiple types of CP according to the nature of injury and area of involvement, it involves posture and movement impairment of cerebral origin, hemiplegic cerebral palsy being one of many of its types.

The rehabilitation of cerebral palsy is always a challenging for professionals and different therapies are listed in literature which follows multiple theoretical framework. Management strategies are basically designed to improve quality of life, reduce and prevent secondary impairments which limit the movement. Beside traditional therapies several new treatments are now added in management protocol for more precise and appropriate targeted results. Less invasive comparatively new therapies include constraint induced movement therapy (CIMT). The learned non use that occurs by insult to the central nervous system, CIMT is directed to treat it by increasing the use of affected limb in functional activities called massed practice and constraining the less affected limb.^3^

This technique, developed by Edward Taub, applies constraint or restricts through any measure the sound limb in hemiplegic patients rendering it unable to use it. The patient is advised to use affected limb to its maximum, repeatedly and under supervision, hence increasing its capacity. This therapy provides the maximum repetition in functional and daily activities of life and enhances the performance as well as promotes the neural plasticity in brain. The duration of constraint and the overall environment are also important factor for its effectiveness in patients with cerebral palsy.^4^-^7^

Another emerging protocol for the management of hemiplegic CP is bimanual upper extremity training. This technique involves task performance using affected limb along with less affected limb in symmetrical or alternating movement pattern which simulates most of our daily activities. This approach gets the benefit of inter coupling of limbs and promote the activity in less affected limb through the sound limb. The Bimanual approach is very crucial in managing the heavy task and where there is need of both limb involvements for attaining the task completion.^8^

Literature shows effectiveness of these strategies. However conflicting evidence is present on which one of these is more effective and no final conclusion can be made to date. This research intends to add to the literature and contribute in reaching final conclusion about superiority of either intervention. This study aimed at determining the effectiveness of constraint induced movement therapy as compared to bimanual therapy for improving functional status in hemiplegic cerebral palsy children. Hypothesis of the study was that “There is difference in the motor function outcome in Constraint induced movement therapy as compared to bimanual therapy in child with hemiplegic Cerebral palsy”.

## METHODS

Total Patients (n = 20) were randomly divided into two groups with treatment group (n = 10) receiving CIMT and control group (n = 10) receiving BMT. This study was conducted in National Institute of Rehabilitation Medicine (NIRM). One child from each group was dropped due to lack of fallow up. Written informed consent was signed by the parents of all the patients. The study design was approved by research ethical committee of Riphah international university, Islamabad. The children with spastic hemiplegia, age between 1.5 and 12 year and having 10 degrees of wrist extension and 10 degrees of finger extension were included in study. CIMT group received personalized ADL task training of affected limb two hours a day, six days a week, for two weeks. Restraint was given for six hours of the waking period. BMT group received personalized ADL task training involving both limbs with same treatment sessions.

This study utilized home setting for providing therapy to CP hemiplegic children owing to the fact that working in natural environment can offer better outcomes by using repertoire of activities and challenges of real world and it can also help cut the treatment cost. Parents were guided initially by the therapist about the timing of constraint and the intervention to be applied. Parents were responsible for their child’s adherence and completion to the treatment program. Through phone contact was maintained with the parents to ensure the progress and protocol adherence.

Type of restraint applied was a mitt that was constraining the hand and the elbow was constrained by sling strapped to the trunk. The personalized activities that were given to the patients of both groups comprised a number of items of daily activities, unimanual activities for the CIMT group and bimanual activities for the BMT group. The tasks to be practiced were comprised of upper limb reach, grasp, manipulation and releasing activity and also bearing weight on the upper limb.

Tool that was used for data collection is the QUEST quality of the upper extremity skills test. It is criterions referenced measure with good reliability.^9^ It measures four functional abilities including dissociated movement, grasp, weight bearing and protective extension. It was made sure that all the patients being included lie on same base level. Score range for patients to be included was between 40 and 60 of grasp and dissociated movement items on QUEST. However for rest of the two items, weight bearing and protective extension, any strict criteria was not established as setting the criteria for more finer items, dissociated movements and grasp, will consequently provide scores accordingly for weight bearing and protective extension. Scores of the QUEST outcome tool were taken by asking the patient to perform activity; according to the activity performed, it was marked tick or cross on the questionnaire. Later on ticks and crosses were counted and put in the formulas given in the manual. The digit provided after that ranges between 1 and 100, with 1 being the lowest and the 100 being the highest level of performance.

## RESULTS

There was a total of 18 patients, nine in each group. The age of the patients was (8.75±3.06). Out of 18 patients, 15 (83%) were male and 3 (16%) were female.

After applying treatment for two weeks post treatment scores were taken. The total score (Table-I) showed significant difference (p < 0.05) and also for dissociated movements and grasp (Table-II). While there is insignificant (p>0.05) difference of weight bearing and protective extension items (Table-III). Dissociated movements and grasp are significantly improved in CIMT treated group as compared to BMT treated group. But there is no difference for weight bearing and protective extension in both groups.

**Table-I:** Mean standard deviation and p value of QUEST total score, pre and post treatment.

Variable	CIMT (n=9) (Mean ± SD)	BMT (n=9) (Mean ± SD)	P value
Pre-QUEST total score	63.05±5.28	61.27±3.68	0.421
Post-QUEST total score	84.12±3.32	79.97±2.23	0.007

**Table-II:** Mean, standard deviation and p value of QUEST dissociated movement and grasp, pre and post treatment.

Variable	CIMT (n=9) (Mean ±SD)	BMT (n=9) (Mean ±SD)	P value
Pre-DM	52.41±8.14	50.43±7.37	0.597
Post-DM	85.91±3.12	82.71±2.47	0.028
Pre- Grasp	53.13±7.20	52.10±5.87	0.743
Post-grasp	87.90±3.13	83.00±3.21	0.005

**Table-III:** Mean standard deviation and p value of QUEST weight bearing and protective extension, pre and post treatment.

Variable	CIMT n=10 (Mean ±SD)	BMT n=10 (Mean ±SD)	P value
Pre-weight bearing	72.97±6.96	70.42±6.87	0.446
Post-weight bearing	81.86±7.78	75.36±6.91	0.080
Pre-protective extension	73.69±6.18	72.15±6.07	0.603
Post-protective extension	80.80±3.25	78.80±2.24	0.149

## DISCUSSION

This study suggested that CIMT is effective in improving grasp and dissociated movement items of outcome tool while there was no superiority observed of CIMT over BMT in weight bearing and protective extension items. In literature there are many studies conducted on the effectiveness of CIMT. A study evaluated mCIMT efficacy on QUEST outcome tool. As concluded ‘Change in mean score of QUEST total score’ was presented with significant improvements.^10^ A study done to compare CIMT and BMT, specificity of intervention showed CIMT to be more effective in unimanual abilities and BMT in bimanual performance.^11^ Another study conducted to compare six hour training to the three hour training of CIMT for maintaining post treatment effects on 6 month follow-up, concluded with no significant difference, and both six hour and three hour interventions being equally effective.^12^ A study was conducted to find out whether combined mCIMT-BMT technique provides any outcome in spontaneous hand use in daily living activities. It found mCIMT followed by BMT to be effective in improving spontaneous use of affected hand.^13^

Grasp and dissociated movements items solely assess unilateral hand ability while weight bearing and protective extension involve the use of both hands. It is inferred thus, there exists specificity of training with CIMT providing better outcome on unilateral function and BMT improving more in bimanual activities. A study done by Sakzewksi L and colleagues in 2011, presented conclusion with findings showing specificity of training, with more unilateral gains in activities with CIMT and more bilateral gains in performance with BMT. Another study conducted by Facchin and colleagues in 2011 concluded with the finding that type of treatment given whether unilateral or bilateral is specific in providing respective outcomes.^11^,^14^

During this treatment home based setting was used to implement the protocol. Literature finds Home based model effective as well. Eliasson 2005, Lin KC 2011, Al-Oraibi S 2011, in their studies supported home based model as a practical alternate to clinical administration of therapy.^15^-^17^ Eliasson AC in 2005, found in his study the patients with low initial scores responded better to CIMT than patients with high initial scores.^17^-^19^

The impairments which were assessed in this study include abnormal posture and range of motion. Sakzewksi and colleagues considered this factor and concluded that activity focused treatment can provide with significant results in functional gains in the absence of any improvement in impairment.^11^

Facchin conducted study to compare CIMT and BMT and concluded that grasping ability is responsive more to CIMT than BMT. A study conducted to find out CIMT effectiveness, concluded that CIMT was more effective in improving grasp than the controlled group and also, grasp showing retention of the treatment effect at 6 month follow-up. Another study also reported same results showing higher scores in grasping as assessed on Peabody Developmental Motor Scale.^14^,^16^,^20^

This study has some limitations of small sample size and short time duration. This study also lacked supervised constraint activity. As such further studies with larger sample size and increased time duration are recommended. In addition supervised constraint activity environment should be used.

Literature shows effectiveness of both of these comparatively newly emerged techniques CIMT and BMT. This study investigated which of the two techniques is better in terms of providing functional outcome as there is conflicting evidence present in literature about superiority of either intervention. This study ended up finding specificity in functional outcome of both training techniques and therefore recommending for future studies to consider combination approach.

## CONCLUSION

CIMT approach is better in improving functional status of child with cerebral palsy as compared to BMT. Significant improvement in grasp and dissociated movement is noted in group of CIMT as compared to BMT in children with cerebral palsy while there was no significant improvement in weight bearing and protective extension in CIMT group when compared to BMT. CIMT is considered the appropriate treatment approach for unilateral conditions while BMT for bilateral conditions.
